# Psychiatry and the Sociology of Novelty: Negotiating the US National Institute of Mental Health “Research Domain Criteria” (RDoC)

**DOI:** 10.1177/0162243919841693

**Published:** 2019-04-12

**Authors:** Martyn Pickersgill

**Affiliations:** 1 Centre for Biomedicine, Self and Society, University of Edinburgh, Edinburgh, United Kingdom

**Keywords:** psychiatry, neuroscience, mental health, novelty

## Abstract

In the United States, the National Institute of Mental Health (NIMH) is seeking to encourage researchers to move away from diagnostic tools like the *Diagnostic and Statistical Manual of Mental Disorders* (the *DSM*). A key mechanism for this is the “Research Domain Criteria” (RDoC) initiative, closely associated with former NIMH Director Thomas Insel. This article examines how key figures in US (and UK) psychiatry construct the purpose, nature, and implications of the ambiguous RDoC project; that is, how its novelty is constituted through discourse. In this paper, I explore and analyze these actors’ accounts of what is new, important, or (un)desirable about RDoC, demonstrating how they are constituted through institutional context and personal affects. In my interviews with mental health opinion leaders, RDoC is presented as overly reliant on neurobiological epistemologies, distant from clinical imaginaries and imperatives, and introduced in a top-down manner inconsistent with the professional norms of scientific research. Ultimately, the article aims to add empirical depth to current understandings about the epistemological and ontological politics of contemporary (US) psychiatry and to contribute to science and technology studies (STS) debates about “the new” in technoscience. Accordingly, I use discussions about RDoC as a case study in the sociology of novelty.

As Keating and Cambrosio (2004, 357) have reflected, the “idea of reducing pathology to biology has an extensive history.” In psychiatry, many have supported the application of biological approaches to comprehending and treating mental ill-health. In the United States, for instance, the National Institute of Mental Health (NIMH) has—alongside its attention to the social and psychological dimensions of purported psychopathology (Pickersgill 2010)—long presented biological approaches to psychiatry as epistemologically “revelatory” (Casey 2017, 239). An emphasis on biology in psychiatry is often linked to the American Psychiatric Association (APA)’s *Diagnostic and Statistical Manual of Mental Disorders* (*DSM*). Detailing all the conditions the APA recognizes as legitimate, the influential 1980 third edition (*DSM-III*) helped to shift attention toward the biological aspects of mental ill-health (Mayes and Horwitz 2005). Subsequently, the *DSM* has become one of the key means through which psychopathology is defined, framed, and acted upon. In the United States and several other nations, it acts as a vital “connective tissue” (Lakoff 2005, 13), materializing and facilitating associations between diverse institutions and actors with a stake in mental ill-health. This includes the APA and the NIMH; for example, the latter funded much of the work underpinning *DSM-III* (Decker 2013) and sponsored planning meetings for more recent DSM revisions (Pickersgill 2014). However, the agency has become more hesitant in endorsing the DSM.

This hesitancy has been instantiated through an NIMH program advocated by former Director Thomas Insel: the Research Domain Criteria (RDoC) initiative. Launched in 2010, RDoC is intended to be a framework for thinking about how specific characteristics of what are deemed psychopathologies can be more precisely investigated in order to produce enhanced understanding and potentially therapeutics (Insel et al. 2010). Insel described RDoC as a project “incorporating genetics, imaging, cognitive science, and other levels of information to lay the foundation for a new classification system” (Insel 2013). It is perhaps more commonly regarded as a neurobiological initiative aiming “to transform psychiatry into an integrative science of psychopathology in which mental illnesses will be defined as involving putative dysfunctions in neural nodes and networks” (Akram and Giordano 2017, 592). In advancing RDoC, the NIMH has downplayed the DSM. As one (in)famous blog post from Insel (2013) described, “Unlike our definitions of ischemic heart disease, lymphoma, or AIDS, the DSM diagnoses are based on a consensus about clusters of clinical symptoms, not any objective laboratory measure.” Consequently, the NIMH would, apparently, “be re-orientating its research away from DSM categories” (Insel 2013).

This article is concerned with how key figures in US (and UK) psychiatry construct the purpose, nature, and implications of the ambiguous RDoC project. Many have offered appraisals of the initiative, including social scientists (e.g., Whooley 2014), in contributions that are variously celebratory and castigating. In particular, RDoC has been called out for its biological emphasis; hence, I want to underscore that my intent here is not to contribute yet another reproving commentary. As indicated, psychiatrists are perfectly able to criticize RDoC themselves and often do so through an idiom similar to that employed by sociologists and others concerned about, for instance, biological reductionism in mental health praxis. Instead of straightforward criticism, I explore and analyze how major institutional actors’ accounts of what is new, important, or (un)desirable about RDoC are constituted through institutional context and personal affects. In so doing, I aim to add empirical depth to current understandings about the epistemological and ontological politics of contemporary (US) psychiatry and to contribute to debates about “the new” in technoscience. Accordingly, I use discussions about RDoC as a case study in what we might term the sociology of novelty.

## Contextualizing RDoC

What is RDoC? Ultimately, it is a kind of epistemic infrastructure providing a framework for (new kinds of) research. RDoC is visualized on the NIMH website as a series of biopsychosocial “domains” (such as “negative valence systems”) that are subdivided into different (generally psychological) “constructs” (https://www.nimh.nih.gov/research-priorities/rdoc/constructs/rdoc-matrix.shtml). One example is “frustrative nonreward,” defined as “Reactions elicited in response to withdrawal/prevention of reward, i.e., by the inability to obtain positive rewards following repeated or sustained efforts” (https://www.nimh.nih.gov/research-priorities/rdoc/constructs/frustrative-nonreward.shtml). “Units of analysis” are detailed for each construct and indicate biological, psychological, and experimental foci for research effects; these disaggregate as genes, molecules, cells, circuits, physiology, behaviors, self-reports, and paradigms. In the case of frustrative nonreward, one paradigm listed is “the Laboratory Temperament Assessment Battery (Lab-TAB),” which is a psychological inventory for assessing “early temperament” in laboratory settings.

The presentation of RDoC on the NIMH website does not lend itself to a straightforward interpretation nor does its description in many publications about the initiative. Rather, elucidating its meaning requires work (and not, as we will later see, only for individuals outside of psychiatry and psychology). Consequently, these meanings multiply as RDoC is discussed in coffee rooms, conferences, blog posts, and journal pages. Nevertheless, RDoC represents a scheme for intervening in the epistemology of psychiatry—and, consequently, understandings of the ontology of psychopathology.

One possible motivation for RDoC is a much-discussed shift away from drug discovery for mental ill-health by the pharmaceutical industry (see Miller 2010). Cuthbert and Insel (2013; who is a key NIMH architect of RDoC) have asserted that psychiatry “has essentially excluded biological findings that do not map on to the current heterogeneous categories of symptom clusters” (p. 3). Accordingly, “issues with the current nosology markedly affect the treatment development arena” (p. 3). Insel (2013) described in his aforementioned blog post that the NIMH was “committed to new and better treatments, but we feel this will only happen by developing a more precise diagnostic system.” Through such public-facing statements, Insel has contributed to structuring the NIMH as a promissory organization (Pollock and Williams 2010) that defines epistemic requirements and generates expectations that these will be provided for (cf. Mittra 2016, 67). In this way, he and other senior NIMH figures consolidate their own authority and that of their organization. This can be read as part of a broader strategy for limiting symbolic and material divestment in (biological) psychiatry and (re)invigorating support for mental health research and development.

RDoC was introduced at a time when the so-called team science was becoming more widely emphasized in mental health research. As in biomedicine more broadly (Mittra 2016), psychiatry is increasingly constituted through large-scale, multi-intuition collaborative projects that entail diverse expertise (Arribas-Ayllon and Bartlett 2014). The Brain Research through Advancing Innovative Neurotechnologies (BRAIN) Initiative is an exemplar. Announced by US President Barack Obama in 2013 as a multidisciplinary, interagency, and private–public partnership, the National Institutes of Health (NIH) (2017) invested US$260 million in the program in 2017 alone through ten of its institutes (including NIMH). As with other neurobiologically focused endeavors, it is imbued with considerable therapeutic promise (Rubin 2008): Insel’s NIMH successor, Joshua Gordon, recently asserted, for example, that “Truly transformative treatments will only come if we invest heavily in basic neuroscience efforts, including but not limited to the BRAIN Initiative” (Society for Neuroscience 2017).

The NIMH has also emphasized the need for, and invested heavily in, whole genomic sequencing (Sanders et al. 2017). It is, for instance, a partner in the Whole Genome Sequencing for Psychiatric Disorders Consortium, which—in the spirit of RDoC—aims to develop a data repository to “facilitate large-scale analyses within and *across* four psychiatric disorders” (Sanders et al. 2017, 1666, emphasis added). Molecular approaches are also supported through core NIMH research platforms, with its Repository and Genomics Resource (NRGR; established in a previous guise in 1998) curating and distributing biomaterials for psychiatric genetic research. As members of the NIMH Office of Genomics Research Coordination have argued, an “explicit goal” of the NRGR “is the mapping of data from traditional DSM-based clinical phenotypic assessments onto RDoC-like domains” (Senthil et al. 2017, 1660). NIMH endeavors like the NRGR reinscribe molecular ways of thinking within psychiatry (Rose 2001). Rhetorically and practically, they also gradually dilute the singular salience of the DSM to psychiatric knowledge production.

Despite such initiatives, it is worth highlighting that the DSM continues to stand in for many as the final arbiter between normality and pathology. This was strikingly visible over 2017, within extensive professional and public debates around whether US President Donald Trump has “narcissistic personality disorder.” Through anchoring discussions in the idiom and categorizations of the DSM, commentators perform and underscore its centrality, authority, and veracity. Still, these descriptors of the APA text are increasingly rendered problematic: it is not uncommon to see articles in prestigious psychiatry and neurobiology journals describing the DSM criteria as, for example, “somewhat ‘fuzzy’ and imprecise” (Braff 2017, 1657). Although the extent to which the DSM adequately captures reality has long been debated (Pickersgill 2014), it is noteworthy that rejecting its facticity and utility is increasingly legitimate within biomedicine.

## Analyzing RDoC

This article analyzes how key mental health researchers and practitioners negotiate the meaning and standing of RDoC as a novel object. Webster (drawing on Barry 2001) has argued that it can be “difficult to identify novelty *in-itself*, since there is much evidence that shows how the same technoscience can be positioned and repositioned as old and new, depending on the networks and audiences it seeks to embrace or mobilise” (Webster 2005, 236, emphasis in original; see also Webster 2002). I take seriously and extend this insight (Pickersgill 2013), regarding novelty not as quintessence but rather as negotiated and attributed through often geographically or institutionally specific forms of sociotechnical praxis. Accordingly, my perspective has some theoretical resonance with other work that has considered adjectival concepts such as analyses of complexity (e.g., Arribas-Ayllon, Bartlett, and Feathersone 2010; Broer, Bal, and Pickersgill 2017; Dan-Cohen 2016).

More generally, I take cues from the methodological relativism orienting early studies in the sociology of scientific knowledge (SSK; Bloor [1976 ] 1991) and related endeavors. In this respect, I am less interested in whether RDoC is “really” novel or not, than in the question of whether and how it is described as such, and with what ramifications. Nevertheless, while I owe intellectual debts to writers like Bloor (as well as, of course, to other foundational and contemporary work in science and technology studies), this paper is not an attempt to develop an explicitly or narrowly SSK analytics of novelty. This not least given the “blind spots” (Delamont 1987) that can result from close adherence to an SSK approach.

This article uses data from, primarily, sixteen semistructured interviews with very influential scientists and clinicians with the professional capital and institutional capacity to contribute in key ways to shaping the contexts of US (and UK) psychiatry. Of these, eleven were themselves certified psychiatrists. The data were collected as part of a wider study exploring changing understandings of diagnostic categories in research and clinical practice within the United States and United Kingdom. The respondents discussed here were selected on the basis of their capacity to influence change in psychiatric praxis more broadly, that is, on account of their institutional position or due to being widely and internationally recognized research leaders.^1^ The sample included current and former senior officials of the APA and NIMH (and other funding organizations), editors of high-impact psychiatric journals, and leaders of major US psychiatry departments (referred to below as R1, R2,…, R16).

In the interviews, I queried participants about their wider clinical and research foci, the place and role of diagnosis in psychiatry, and their experiences and perspectives regarded RDoC. My respondents spoke with various degrees of frankness; one asked midway through the interview for the voice recorder to be paused, while others communicated through tone and facial expression that which they were cautious about stating on tape. Following transcription, the data were thematically analyzed, with interviews coded according to the initial concerns of the research and issues that became apparent as the study progressed.

In what follows, I present and discuss these data, describing different ways through which the novelty of RDoC was constructed and negotiated. I highlight, in particular, reflections on the kinds of research RDoC might propel, concerns about the distance between the laboratory and the clinic the initiative could expand, and antipathy expressed toward it, to the NIMH, and to Thomas Insel himself.

## Epistemic Catalysis

Commenting on the introduction of RDoC, R4—a senior member of the NIMH—reflected, “at first it was very novel to people, very different from how most people had thought about these disorders.” Initially, R4 felt that many “traditional psychiatrists had a hard time thinking about the disorders in any but the DSM categories.” As he puts it: “It’s like ‘what do you mean there’s no depression and no schizophrenia and I have to think about these other things?!’ and that was a hard shift.” Nevertheless, “many psychiatrists, and psychologists, who do neuro-imaging and those types of areas naturally felt like ‘good, this is the opportunity to really integrate these things.’” R4 described how RDoC was “welcomed”: those “who had been saying, ‘you guys need to do something different about diagnosis,’ they said ‘good, this might not be the way we thought about it but we’ll move that way.’” Today, R4 felt, “people have caught on to the idea” of RDoC; there is a “quite healthy groundswell or even accelerating, you know, tide of people saying that this is really how we need to go.”

Certainly, several of my respondents indicated degrees of approval of RDoC as a stimulus for new work in, primarily, biological psychiatry. In this respect, RDoC was presented as a “very interesting” way of “addressing research problems” (R1), a worthwhile “experiment” (R13), and an “exciting” (R14) and “very courageous” initiative that could offer “a huge cultural change” (R3). Some respondents discussed how this work would enhance “treatment development” (R12), which R3—editor of a major psychiatric journal—claimed was “part of the reason people got interested in moving toward RDoC.” For him, “as we, get closer and closer to the cellular pathology of the disorders we are going to discover, I would predict, that our treatments are more and more specific.” Part of the novelty of RDoC, then, was its purported capacity to propel biomedical innovation.

In one of the longer interviews, the enthusiastic R3 told me over lunch that various different aetiologies for the category of depression exist. These, he asserted, were treated as if they “are all the same”—but “they’re *not all the same*.”^2^ This kind of position about the ontology of pathology is increasingly evident within the psychiatric literature and has been apparent in many of my ongoing conversations with scientists and clinicians. R6 described how RDoC spoke to the kinds of complex ontological imaginaries that are now finding increased traction:

Psychiatric research, because of the DSM, and other diagnostic systems, has been focused on disease entities, these so-called polythetic constructs. Where a, a erm multiple permutations of signs and symptoms can give rise to one particular diagnosis, depression being a good example. And the idea was that, it doesn’t really matter how we come up with these different clusters, as long as they are being reliably diagnosed, they will lead to some common pathway, to some common pathology. And RDoC simply states that that’s unlikely going to be true, because we haven’t found very much with that strategy for the last decades. And to really, that’s really the issue, to benefit from the power of neuroscience and genetics research, to really maximize the potential impact of basic science research, we need to now *reframe* the research in psychiatry. We need to refocus it away from the psychiatric diagnosis.

In the extract above, we can see how R6’s account of the conceptual underpinnings of RDoC explicitly contrasts these with those of the DSM. This kind of comparative account as a means of signaling the import and novelty of RDoC is currently common in clinical psychiatric and neurogenetic discourse. For instance, R8—a key member of a UK funding agency—stated that one advantage of RDoC was that “it tried to move away from DSM-defined diagnoses into breaking it down into more mechanistic phenotypes.” A former leader within the NIMH, R12, also cast his discussion of RDoC in relation to the DSM (and in the strongest terms of all the respondents). For him, “the main advance” of RDoC was “liberating investigators from the cognitive tyranny of the DSM categories.” Even R1, a senior member of the APA critical of the dimensionality of RDoC and a defender of the categorical approach of DSM, noted the challenges of researching DSM disorders rather than specific components of those constructs:

one thing that I think anybody who knows something about this will agree, is that the categories that we use in psychiatry are not monolithic, they *probably* are, you know, amalgams of several sub-categories. And to the extent that that’s true, that makes it a lot more difficult to identify the biological underpinnings.

Appraisals of RDoC were thus commonly grounded in characterizations of what the participants felt psychiatric pathologies *really* were and the degree to which research that employed the DSM system could accommodate such ontologies. Accordingly, for US-based interviewees (who generally had much to say about RDoC, both good and bad), the novelty and significance attributed to RDoC related to its perceived conceptual distance from the DSM. This underscored the extent to which the DSM acts, still, as a conceptual “anchor” (cf. van der Sluijs et al. 1998) within US psychiatry.

In the United Kingdom, however, the DSM is less central to mental health praxis. Although influential, the World Health Organization’s International Classification of Diseases is more significant within clinical infrastructures (e.g., through the National Health Service information technology systems). Further, few scientists would see the NIMH as a go-to funder; rather, the Wellcome Trust, the Medical Research Council, and the UK Department of Health and Social Care’s National Institute for Health Research are the key mental health research sponsors. This context shaped the accounts of my UK interviewees. Generally, RDoC was presented as having salience for US researchers applying to the NIMH and interested in DSM diagnostic entities but as less (or ir)relevant to UK scientists seeking domestic sponsorship.^3^ For instance, R16, an eminent UK psychiatrist, noted that since he was not in receipt of NIH funds, RDoC was “not worth bothering about” (although he had some strong views about its relationship to [US] clinical psychiatry, as we will later see). While RDoC emerged as part of the NIMH landscape in 2010 (Insel et al. 2010), when I asked one journal editor, R7, how aware UK psychiatrists were of RDoC prior to Insel’s infamous 2013 blog post, he replied: “Oh I don’t think they were very aware of it at all.” In response to a query about his position on RDoC, R17, a senior member of the Royal College of Psychiatrists, told me:

I mean I have the same view as anyone, I’m sure one day it’ll work, but it’s, it was a bit premature. And I mean, we’ve had those promises before, so far they haven’t, I mean, it’s a bit like the whole DSM-5 rhetoric itself, it was ahead of itself. There’s nothing wrong with those ideas, it’s just they’re not there yet, so unfortunately the whole thing turned out to be a bit of a damp squib, didn’t it? And er so, yeah. I mean I know Tom Insel, and he’s a very bright guy, but I mean I think he’s struggling the same way everyone else is. One day there’ll be these changes, but they haven’t yet really happened, and we just continue to work away as we do.

R17’s talk minimized the import of RDoC, presenting it as a worthy enough initiative that was nevertheless less impressive than intended (i.e., “a damp squib”^4^). An absence of import was not necessarily problematic, however: researchers and clinicians would “just continue to work away” as they have always done. In many of the comments from US-based professionals, the counterposing of RDoC and the DSM as different approaches to understanding mental ill-health presented RDoC as self-evidently novel; yet, the comments of R7—made in an institutional context where neither the NIMH nor the APA hold any kind of formal influence—unsettle framings of innovativeness as such.

## Clinical Distance

While some participants enthusiastically discussed the epistemic catalysis RDoC might enjoin, others—and occasionally the same interviewees—expressed disquiet about its conceptual underpinnings and implications. When I ventured in an interview with R2, a prominent US scientist, that I personally found the RDoC matrix a little confusing, they replied: “I think it’s really confusing, particularly to clinicians.” R2 reflected that “unfortunately what has happened is we have gone from diagnosis to a new boxology”—the “simplicity” of which has “hindered our science.” Ultimately, the RDoC matrix was “just kind of a ridiculous thing.” This ridiculousness was partly related to the institutional cartography R2 felt was implied by RDoC, wherein clinical and basic research were positioned as far apart:

every single RDoC RFA [Request for Applications], that has come out of the United States has basically said, animal—if you’re combining animal studies with this, need not apply to this mechanism. Which, to me […] is shit, because the *only* way that we can really understand mechanisms is if, if we can really work with the animal scientist. And *just* when I felt like clinicians and basic scientists, both human and animal, were coming together, this pushed us into a silo again.

The imaginaries of clinicians that contoured R2’s reflections manifested in many of my discussions with mental health scientists (some of whom drew on their own clinical experience and expertise to adjudicate RDoC). In a minority of interviews, characterizations of clinicians that emphasized their epistemic limitations were leveraged as a mechanism to explain a lack of practical salience for RDoC. R3, for example, was personally positive about RDoC, but noted challenges for other psychiatrists who had “I would say an amateur’s understanding of the brain.” Consequently, it was “hard to imagine” how psychiatry might move toward a conception of pathology that was “more nuanced and realistic and brain based.” In R6’s words, most US psychiatrists “feel they can be a good enough” without “really knowing anything about the brain”; hence, nothing enjoined them to engage with RDoC or the work it sought to propel.

Far more common than castigations of clinicians, though, were direct criticisms of the design of RDoC and of NIMH funding emphases. It was these that the majority of my interlocutors constructed as generative of the purported distance of RDoC from the clinic, rather than a failure of imagination or training on the part of psychiatrists. For instance, despite describing RDoC as “interesting” for research purposes, R1 stated that “as someone who knows quite a bit about the RDoC project, I can tell you that it’s *so not ready* for prime-time it’s not funny.” In particular, it did not “comport well with how doctors in general are trained or think about things.” R10, a senior psychiatrist, similarly noted that RDoC “doesn’t start out with patients as ordinary doctors would identify them, and that’s a real problem.” One journal editor, R7, also described how RDoC—despite being “interesting”—“suffers *I think* from not really having much to do with the clinic right now.” R9—a very influential US-based researcher—explained this disconnect between the epistemic ambitions of the RDoC initiative and its clinical ramifications through the origins of the RDoC initiative: “RDoC was designed kind of in an ivory tower to try to improve conceptualization of research. And it wasn’t at *all* designed thinking of the day-to-day activities of [psychiatrists].” Its limitations were unpacked as follows:

RDoC certainly has some virtues. But it doesn’t translate at all well to what the average psychiatrist does when he or she sees a patient. I mean, they’re not going to say “does this patient have a problem with positive valence systems?” They’re just not. Or negative valence systems or, you know, whatever one you want to pick. They’re going to see people coming in. They’re used to asking about, you know, how was their mood and how was their sleep and how was, you know, all the various things that go into evaluating a patient and making a diagnosis. And, it’s going to be a long way from RDoC to daily clinical practice.

In the UK, R16 noted that “it’s quite good that we have alternatives” to the DSM, but he did not particularly “like the RDoC” since it “didn’t seem to make much clinical sense to me.” Like R9 above, he drew attention to the lack of clinical expertise enrolled in the development of RDoC: “there were no practicing clinicians on the committee that drew it up. So it’s I think in my view over, over influenced by mouse models and so on.” Although R16’s own research was quite biologically oriented, he was also critical of the apparent somatic emphasis of RDoC: “I think it’s been too, it’s been a hundred per cent biological, and it operates on the basis that psychiatric disorders are a manifestation of brain pathology or brain deviance. So, I’ve been a bit hostile er to that.”

Resonant with such perspectives, R6 asserted that the RDoC initiative was “basically a revolution of cognitive neuroscientists who have now seen the opportunity to shape some of the psychiatry research according to their view of the world. And that will go on for a little while, until people realize it will not lead to anything in psychiatry.” Ultimately:

I will go so far to say that when everything is said and done, RDoC will be a great boost for new knowledge in neuroscience, with little impact on psychiatry. It will lead to a significant advance in our understanding of human behavior, and the neural basis of human behaviors. […] But it will have little impact on the true task of psychiatry.

The “true task” of psychiatry would only be ensured if the introduction of RDoC had tangible consequences: particularly, though not exclusively, the development of new therapies. Yet, most interviewees seemed to regard this as a very distant prospect. R11, for instance, claimed that RDoC “is probably not going to yield anything for a *long* long time, in terms of new treatments.” R9 likewise asserted that they had “thought quite a bit about RDoC” and could not “*see it* translating to treatment.” When I asked R1 whether treatment development might link with RDoC in some way, their response underscored widely perceived institutional and conceptual complications:

It’s a very good question, certainly for pharma, the determining factor usually is the FDA, in terms of how they design their trials, and it would be difficult to imagine the FDA would move away from the current model that it has, where medicines are for indications, erm diagnostic indications generally speaking. So erm it’s hard to imagine that they would move away from that. In particular, since we don’t even have a sense of, like as an example, erm if you’re looking at constructs that I have a very hard time with is, frustrative nonreward. Don’t ask me what it means, I’ve read it many times, I don’t understand it. But we don’t have a threshold beyond which we, we think its pathological to have that, so then how would you determine when you institute a treatment? You know? There’s just not enough information.

In the comments above, R1 moves almost seamlessly from a relatively dispassionate assessment of RDoC and innovation in terms of the path-dependencies associated with the Food and Drug Administration to a more affective critique of RDoC constructs. Movement across critical registers was not uncommon within the interviews; in particular, participants skipped between complaints about RDoC, negative assessments of the NIMH, and judgments about Thomas Insel himself. With regard to the latter, criticism was sometimes muted or mild. For instance, R5 (a senior researcher) noted that RDoC was oriented toward “the research community” not “at *practitioners*”; by remarking that “I think Insel would admit that,” R5 implied this was a weakness. However, as we will now see, far more explicit (and imbricated) criticisms of Insel, RDoC, and the NIMH were also evident.

## Against Unilateralism

The extent to which RDoC had ruffled the feathers of many in US psychiatry was highlighted in my interviews with UK psychiatrists and funders. As R16, an influential UK psychiatrist, described:

I was quite happy to see Tom Insel arguing with the DSM-5 people but I…I think, RDoC got a lot more attention because Tom Insel controlled the funding. And I, a lot of American researchers were privately very hostile to it but they knew that you couldn’t get funded unless you went-went along with it.

I likewise encountered hostility in my encounters with US clinicians and researchers. Notwithstanding the various critical commentaries that had been produced about RDoC, I was still a little surprised at the extent of this. Some reactions were couched in terms of pragmatic self-interest, with interviewees sharing their own and others’ concerns that RDoC had negative funding implications based on how they had studied psychiatric disorders to-date. Others, as we will see, related more to the particular set of relationships of authority that enabled, and were consolidated through, the ways in which RDoC was presented to the (US) research community.

Early in my research, I spoke with R4, a senior member of the NIMH. As detailed above, he described what he saw as a growing interest in RDoC from scientists especially. When I asked about the wider reception to RDoC, he noted a series of “misperceptions.” Conjuring a kind of deficit model of psychiatric understanding of the initiative, the NIMH, and Insel, R4 ultimately seemed to feel that fuller comprehension of RDoC would lead to more widespread acceptance of it. In fact, some psychiatrists appear to have responded more, not less, negatively to RDoC as the initiative has unfolded. R9 puts it this way: “I guess when RDoC first appeared I felt better about it than I do now.” The initiative was deemed to have “become a primary goal of NIMH to the exclusion of other ways of thinking,” and “even though it looks complicated, it’s oversimplified things.”

For R4, one misunderstanding about RDoC was that it “seems very prescriptive to people.” However, he felt its true purpose was “*just the opposite*”: “it’s to help us free up people.” Still, R4’s view that RDoC appeared “prescriptive” was certainly corroborated by my interviews. R10, for instance, told me that “There’s been a *lot* of concern, particularly in the clinical research community, about the Director of the NIMH” and noted that RDoC had been “divisive.” When I asked what that meant, he replied: “in the sense that there were a lot of people in the clinical research community who felt that this was something that was being *imposed* on, rather than *deriving from* the material. You know, the scientific material. That there was a kind of artificial imposition.” R6, editor of a prestigious psychiatry journal, similarly reflected:

the fundamental flaw of RDoC is that it was made as an executive decision. It was made from a group of people that were able to *control* the allocation of resources. And researchers typically do not er look fondly on such a[n] *executive decision* to shape how they should think, you know how they should do their work.

One high-profile psychiatrist, R5, described how Insel “has been *far* more proactive in shaping research agendas than previous directors, thinking that he knows the right way for psychiatric research.” To this end:

What Insel has done is basically taken, if we do a run from very basic molecular cellular erm small circuit kind of *large network* imaging neuropsychology, what he’s basically said is *that patch* is where we’re going to get all the action. So it’s really the sort of marriage of function—of systems of neuroscience and neuropsychology. And that’s where we’re going to really find answers. *Wow*! That’s a fair touch of hubris.

Through RDoC, R5 felt the NIMH were “asking interesting questions, so if this were a recommendation that people might use, I wouldn’t have any problem with that. It’s really the enforcement part that I take umbrage at.” In contrast to Insel’s style of leadership, he argued that it was not “the goal of funders at the level of directors to be *highly* proscriptive.” This, he felt, was “*not* their job.” Insel, though, had “reached down and shifted resources in a more forthright way than I actually think is optimally healthy. It’s actually a little scary.”

Two representatives of UK funding agencies I spoke with shared the view that Insel had acted somewhat unilaterally over the release of RDoC. While one (R8) noted that he had been “very smart” and had “pushed people” to shift their research emphases, another (R15) said:

I don’t think, I don’t see any move, at least at the moment, for us to go as far as NIMH who have sort of *mandated* that the research *has* to go you know under the research domain criteria *rather than* diagnosis-based. We’ve kind of got flexibility there. I don’t think we would probably force people to go down that route.

Much of the critique emerged in the interviews after I asked about wider reactions to the director’s blog post quoted in the introduction to this article. Part of the furor about this related to its release just a few weeks before publication of the much-anticipated *DSM*-5. My respondent at the NIMH (R4) noted that this time line “looked like [Insel] had deliberately timed it,” though claimed this “wasn’t so.” R1 laughingly recalled the piece as “uncharacteristically intemperate!” R2 felt that “it kind of said that we don’t know anything about mental illness and didn’t have a biological base. I mean, it can be misinterpreted that way.” This “upset” a “lot of people.” R11 told me the following story when I asked about whether there had been much discussion about the blog post:

[W]e have a weekly lunch meeting. That was really the hot topic. Er, erm, I think there was, er [sigh]. At the time, I think there was a lot of criticism of Tom because he failed to recognize […] that there are a lot of therapeutic approaches that exist that really help people. And none of them er were discovered by any kind of scientific process. Erm, that if we gave up on trial and error and serendipity, we’d have nothing for patients and, don’t you care about patients? I mean, I think that was a lot of the er blowback, you know, on this side, is this, this was an anti-therapeutic stance that was also unrealistic and not consistent with reality. It was aspirational, and maybe erm something to guide us going forward, but to erm er *demean* what exists as if people aren’t helped, we know they are helped. Again, it’s maybe a process that’s partly art and partly evidence informed and partly trial and error. But a lot of people depend on and a lot of people get help. And his, the way he er presented, I don’t remember it that clearly, but I remember generally, presented it was the, *demeaning* of that process.

As with R2, then, R11 described he felt how the blog post sold psychiatric knowledge and practice short and reacted strongly against this purported claim. R9 responded similarly:

He spoke too impetuously or without adequate caution, in terms of thinking about the consequences of what he was saying in terms of *patient* perceptions, family perceptions. And so he, you know, made those bold, very negative statements and then he realized, I mean, a number of people gave him feedback and he realized he should backtrack a little, but the damage had already been done. And to be the director of the National Institute of Mental Health that’s a bully pulpit. I mean, the president of the APA, the head of NIMH, those are probably the two *biggest pulpits*, and even you know major journals […] don’t have that level of influence. They may have lesser levels so for Tom to er be *so negative* about the day to day activities of, you know, *thousands* of psychiatrists has had a bad impact on them, on patients, and on families.

R9 felt that the antipathy toward Insel was widespread:

I can tell you that since Tom has decided to leave nearly *everyone* that I’ve talked to has said something that they didn’t feel they could say when he was still there which was that he has done more damage to clinical psychiatry than can be repaired in five or ten years.

Indeed, they were “astounded” by “how many people I’ve ran into since he decided to leave who have said, ‘*Gosh.* What a relief.’”

In sum, RDoC was presented in my interviews as discordant with established norms for leveraging disciplinary innovation: that is, its introduction was seen as an “executive decision” made by Insel. This discordance was not generally received well by the US clinicians and researchers I spoke with; rather, it channeled a range of negative affects that bled into and hence connected appraisals of RDoC, NIMH, and Insel. Thus, critiques of RDoC were very commonly advanced as sometimes strong criticisms of Thomas Insel. Whether or not RDoC could be understood to be stimulating innovative psychiatric research underpinned by new ontogenies of psychopathology was, it seems, as or even less salient than the fact that the initiative was perceived as packaged and prescribed in the absence of wider consultation with the (US) mental health community. A significant feature of the novelty of RDoC constructed in the interviews, then, was its mode of instantiation.

## Discussion and Conclusions

In this article, I have analyzed how key figures in US (and UK) psychiatry construct the purpose, nature, and implications of the NIMH RDoC project. The perspectives of my respondents were not homogenous; in particular, differences in the appraisals of RDoC between US and UK interviewees indicate how novelty is socially located and produced, rather than directly reflecting some intrinsic property of an ostensibly new entity. In many areas of the humanities and social sciences, it remains common to take scientific designations of originality at face value and then to advance analyses of the social, legal, and ethical ramifications of an ostensibly novel entity or practice. In contrast, my analysis underscores the need to consider novelty as an empirical object in its own right (see also Webster 2002, 2005). The originality of RDoC (or any other purported innovation) is hardly clear-cut, or something that preexists its appraisal; instead, accounts of what is new, important, or (un)desirable are constituted through and with institutional context and individual subjectivities.

Just because novelty requires unpacking does not mean its ascription lacks salience. When the NIH and its subinstitutes (like the NIMH) elect to move their operations forward in particular ways, and perhaps especially when those are characterized as “new,” those they fund need to orientate their work in alignment. As Mittra (2016, 67) notes, “strategic decisions made by organizations like the NIH […] enact particular values and valuation practices that have a material impact on the innovation ecosystem and the practices therein.” Grantees of the NIH, as well as clinical opinion leaders whose professional communities are presented as beneficiaries of the research of sponsored scientists, are all too aware of the effects institutional decision-making can exert on epistemic economies. The sometimes strong statements regarding RDoC, the NIMH, and Thomas Insel that I encountered on and off record in my interviews with leading psychiatrists and psychologists are legible in this light.

The most affectively salient aspect of the novelty of RDoC constructed within the interviews was not so much its conceptual architecture (though this was configured as new), but the relationships between Insel, the NIMH, and the wider (US) psychiatric community that RDoC instantiated. Thomas Insel was widely perceived as acting unilaterally to impose a form of epistemic infrastructure on mental health research, regardless of its alignment with the existing imaginaries and practices of clinicians and scientists. A partial shift in psychiatric emphasis was produced through a much more jolting break from usual modes of enjoinment.

In the US and elsewhere, tensions between the laboratory and the clinic are long-standing and multifaceted (Kraft 2013). Articulations of friction also contribute to constructing distance between these domains, participating in their reification as distinct spaces. RDoC was largely described as conceptually and operationally distant from the clinic, with its novelty and possible utility constructed as primarily relevant to the epistemology of psychiatry—not the therapeutic practice of clinicians. My interlocutors generally advanced low expectations (Gardner et al. 2015) about the potential of the initiative, resonating with other STS accounts of biomedicine that have documented scientific skepticism about the import of biomedical research for patient care (e.g., Wainwright et al. 2006). In advancing low expectations about the therapeutic implications of RDoC, my participants thus challenged its import and implications as rehearsed by senior NIMH officials and performed the distinctiveness and primacy of the clinic within psychiatric praxis. This can itself serve strategic ends within a (US) context in which discovery (neuro)science is prioritized by mental health funders.

When introducing this article, I promised to refrain from advancing my own straightforward criticism of RDoC; I instead sought to underscore how encounters with it dynamically interrelate with wider epistemological, ontological, and affective positions and politics in psychiatry. Such normative abstinence on my part foregrounds the critical perspectives of clinicians and scientists themselves, much of which aligns with social scientific concerns about an NIMH emphasis on neurobiological research (e.g., Whooley 2014). That these criticisms were made by leaders in a profession long taken to task by social scientists and others for its somatic emphasis suggests that it might be time to revisit taken-for-granted assumptions about how psychiatrists (want to) undertake their clinical and scientific work.^5^

## Notes

1. Decisions about whom to contact as institutional and/or research leaders were shaped by my ongoing research in the sociology of mental health, conducted over successive projects since 2005.

2. Emphasis in original (and likewise throughout the paper, unless otherwise stated).

3. That said, during my ongoing fieldwork, I have increasingly found that UK scientists are mentioning Research Domain Criteria in their funding bids.

4. This colloquial phrase has its origins in the lexicon of nineteenth-century coal miners where a “squib” was an explosive device.

5. We might also want to raise questions about the distinctiveness of sociological ontologies vis-à-vis those of psychiatry. As Millard (2017) has shown, the imbrication of psychiatric and sociological thought has long been considerable. Within my own fieldwork, it has been common to see psychiatrists underline social aetiologies of subjective distress; I also frequently encounter social scientific colleagues employing psychiatric and psychological idioms to make sense of their own research findings. What, if anything, this observation implies is beyond the scope of a footnote; but certainly, the history and future of the psychiatry–sociology relationship requires further consideration.
